# Recombinant Human Fab Antibodies Differentially Neutralize Shiga Toxin in Renal Epithelial and Endothelial Cells

**DOI:** 10.3390/toxins18060257

**Published:** 2026-06-05

**Authors:** Fernando D. Gómez, Daniela Luz, Isabel Chinen, Daniel Girón, Raissa L. Ferreira, Camila Henrique, Ariela O. P. Bom, Izabella M. Henrique, Wanderson Marques da Silva, Flavia Sacerdoti, Elizabeth S. Miliwebsky, Gang Chen, Claudia C. Carbonari, Sachdev S. Sidhu, Roxane M. F. Piazza, María Marta Amaral

**Affiliations:** 1 Universidad de Buenos Aires, Facultad de Ciencias Médicas, Departamento de Ciencias Fisiológicas, Laboratorio de Fisiopatogenia, Buenos Aires C1121 ABG, Argentina; gomezfernandod@gmail.com (F.D.G.); claudio.gironr@gmail.com (D.G.); wanderson.marques1@gmail.com (W.M.d.S.); fsacerdoti@fmed.uba.ar (F.S.); 2 CONICET-Universidad de Buenos Aires, Instituto de Fisiología y Biofísica Bernardo Houssay (IFIBIO Houssay), Buenos Aires C1121 ABG, Argentina; 3 Laboratório de Bacteriologia, Instituto Butantan, São Paulo, SP 05503-900, Brazil; daniedaluz@gmail.com (D.L.); raissalozzardo@gmail.com (R.L.F.); camilahpp20@gmail.com (C.H.); ariela.bom.esib@esib.butantan.gov.br (A.O.P.B.); izabellahenrique@gmail.com (I.M.H.); 4 Centro de Desenvolvimento de Anticorpos, Instituto Butantan, São Paulo, SP 05503-900, Brazil; 5 Infectious Hazard Management Unit, Public Health Department, Pan American Health Organization, Washington DC 20037, USA; ichinen05@gmail.com; 6 Servicio Fisiopatogenia, Departamento Bacteriología, Instituto Nacional de Enfermedades Infecciosas ANLIS “Dr. Carlos G. Malbrán”, Buenos Aires C1282 AFF, Argentina; emiliwebsky@gmail.com (E.S.M.); ccarbonari@anlis.gob.ar (C.C.C.); 7 Terrence Donnelly Centre for Cellular and Biomolecular Research, University of Toronto, Toronto, ON M5S 2Z9, Canada; gchen2012@gmail.com (G.C.); sachdev.sidhu@gmail.com (S.S.S.)

**Keywords:** Shiga toxin, STEC, Fabs, hemolytic uremic syndrome, toxin neutralization

## Abstract

Hemolytic Uremic Syndrome (HUS) is a severe clinical manifestation primarily triggered by Shiga toxin-producing *Escherichia coli* (STEC). While Shiga toxins (Stx) are central to the development of systemic endothelial damage, current recombinant antibody developments have overwhelmingly focused on neutralizing the Stx2 subtype. However, numerous STEC isolates produce Stx1 either independently or alongside Stx2, revealing a critical need to diversify the antibody repertoire for comprehensive antitoxin therapies. To address this, we characterized two novel, fully human recombinant Fabs targeting Stx1 (FabB6:Stx1 and FabC8:Stx1) selected from a synthetic library via phage display. We evaluated their binding specificity and neutralizing activity in Vero and human proximal tubular epithelial (HK-2) cells, as well as in primary human glomerular endothelial cells (HGEC exposed to HUS-derived STEC supernatants. Both Fabs exhibited high specificity and nanomolar affinity for Stx1. Notably, they displayed cell-type-dependent neutralization profiles, with FabC8:Stx1 demonstrating superior and more consistent neutralization in HK-2 cells. Crucially, when evaluated alongside previously characterized anti-Stx2 antibodies (FabC11:Stx1/Stx2 and FabF8:Stx2), the Stx1-specific Fabs conferred complementary protection against clinical STEC isolates. These findings support the inclusion of Stx1-targeting recombinant antibodies into broader multi-toxin neutralization strategies, thereby expanding the therapeutic potential against STEC-associated diseases.

## 1. Introduction

Hemolytic Uremic Syndrome (HUS) represents a critical clinical manifestation characterized by the triad of microangiopathy hemolytic anemia, thrombocytopenia, and acute kidney injury [1,2]. This condition is primarily triggered by Shiga toxin-producing *Escherichia coli* (STEC), a global foodborne pathogen that releases potent cytotoxins. These virulence factors, known as Shiga toxins (Stx), are encoded by lysogenic bacteriophages and are central to the development of systemic endothelial damage [3]. Although Stx1 and Stx2 subtypes share a common AB_5_ structural framework and cytotoxic mechanism, they exhibit distinct epidemiological patterns and clinical correlations; notably, Stx2 is more frequently associated with progression to severe HUS [4,5].

To date, a targeted therapeutic agent to neutralize Stx in clinical settings remains unavailable. The use of antibiotics is still a subject of intense debate, as they may trigger enhanced toxin synthesis and release, restricting current management to supportive care for STEC-infected patients and those progressing to HUS [6,7]. In this context, toxin-neutralizing antibodies represent one of the most promising strategies to prevent toxin-mediated endothelial damage and disease progression. Considerable efforts have been devoted to developing antibody-based and other therapeutic approaches targeting Stx [5,8,9]. More recently, a neutralizing equine anti-Stx hyperimmune immunoglobulin F(ab’)2 Fragment preparation INM004 has emerged as a potential immunotherapeutic option for STEC-associated HUS after hospital admission, to prevent long-term renal damage [10]. The successful completion of ongoing phase III clinical trials could enable the earlier and broader use of this therapy for STEC-associated HUS [11].

Compared to conventional monoclonal and polyclonal antibodies, synthetic recombinant antibody fragments selected via phage display offer distinct advantages, including reduced immunogenicity, high specificity, and improved scalability through cost-effective bacterial expression systems.

Using this technology, our group has previously developed and characterized two human recombinant antibody fragments targeting Stx2 (FabC11:Stx1/Stx2 and FabF8:Stx2) [12,13]. Notably, FabC11:Stx1/Stx2 exhibited cross-reactivity with Stx1 and provided potent neutralization and protection against Stx2-mediated toxicity in both murine models and human renal cells [12,13]. Furthermore, FabF8:Stx2, a fully human recombinant fragment that demonstrated high affinity for Stx2, effectively shields human glomerular endothelial cells from apoptosis, detachment, and intracellular edema induced by purified toxins. In addition, FabF8:Stx2 protected Vero cells against STEC supernatant-induced cytotoxicity [14].

Despite significant progress, the development of recombinant antibodies has focused almost exclusively on Stx2. Nevertheless, numerous STEC isolates produce Stx1, either independently or alongside Stx2, and toxin expression profiles may shift throughout the infection cycle. Thus, diversifying the recombinant antibody repertoire to include Stx1-specific candidates is vital for establishing comprehensive antitoxin therapies; however, this approach remains relatively underexplored. Indeed, there are only a few examples in the literature for anti-Stx1 monoclonal antibodies. The chimeric antibody cαStx1 (human/mouse version of 13C4) targets the B subunit of Stx1, blocking receptor binding and toxin internalization. Its neutralizing activity has been demonstrated in Vero cells and Stx1-intoxicated mouse models, with subsequent evaluation in Phase I and II clinical trials [15,16,17]. Additional anti-Stx1 monoclonal antibodies, predominantly directed against the B subunit, have shown effective toxin neutralization in vitro (HeLa cells) and in vivo (mouse models) [18]. Similarly, the monoclonal antibody 5-5B inhibits Stx1 receptor binding and cytotoxicity, as demonstrated in Ramos cells [19].

Therefore, we hypothesized that incorporating Stx1-targeting Fabs would complement existing anti-Stx2 agents, thereby broadening the neutralization spectrum against Shiga toxin-mediated cytotoxicity. Accordingly, the objective of this study was to characterize two novel human recombinant Fabs directed against Stx1, FabB6:Stx1 and FabC8:Stx1, and to evaluate their binding specificity and neutralizing activity, both individually and in comparison with previously characterized anti-Stx FabC11:Stx1/Stx2 and FabF8:Stx2. This integrated approach seeks to provide a robust framework for multi-toxin neutralization, with implications for the clinical management and diagnosis of STEC-related disorders.

## 2. Results

### 2.1. Generation and Characterization of FabB6:Stx1 and FabC8:Stx1

**Figure 1:**
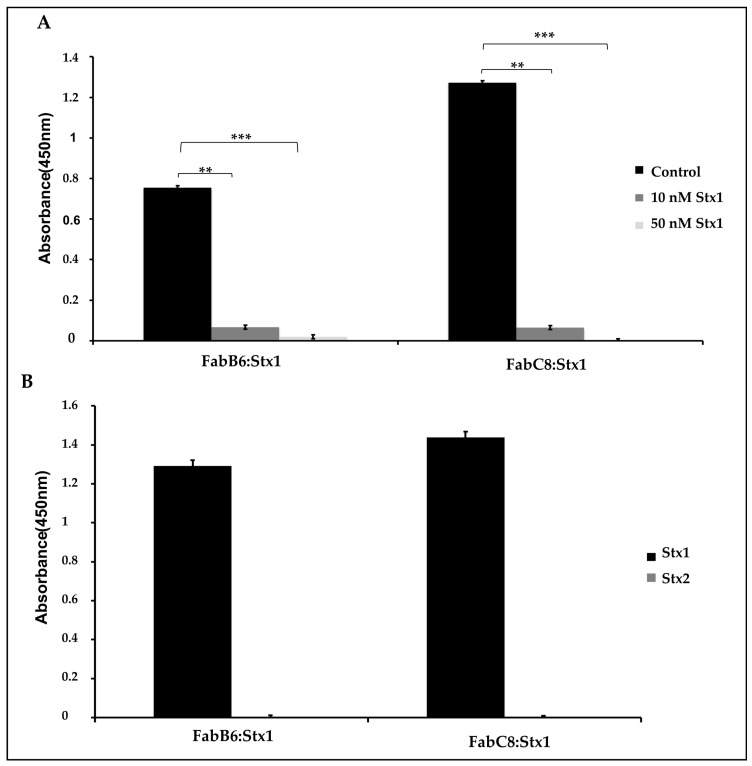
(**A**) Competitive ELISA analysis of phage-displayed Fab clones selected against Stx1. Phages were pre-incubated with soluble antigen at concentrations of 10 or 50 nM before incubation on toxin-coated plates; PBT buffer was used as the negative control. Data are presented as mean values from triplicate assays. Statistical signif-icance relative to the control was determined using Student’s t-test. The Stx1-specific phage clones FabB6:Stx1 and FabC8:Stx1 are shown. ** *p* < 0.01, 10nM Stx1 vs. Control; *** *p* < 0.001, 50 nM Stx1 vs Control. (**B**) ELISA-based cross-reactivity analysis of phage-displayed Fab clones against Stx1 and Stx2. Plates were coated with antigen at 2 μg/mL, and phages were applied at a fivefold dilution. All assays were performed in triplicate.

FabB6:Stx1 and FabC8:Stx1 antibody fragments were selected by phage display from a naive, highly diverse (10^10^ clones) human synthetic Fab library using purified Stx1 as the target antigen. After four rounds of biopanning, 45 phage clones showed detectable binding to Stx1. Sequencing analysis revealed two unique Fab sequences, designated FabB6:Stx1 and FabC8:Stx1. While sharing identical framework regions and identical CDR-L1, CDR-L2, and CDR-H2 loops, the structural differences, following the library design, are primarily localized to the CDR-H3 and CDR-L3 regions, which differ significantly in amino acid composition. Notably, the CDR-H3 also differs greatly in loop length (5 versus 18 amino acids, respectively), and minor variations were also observed in the CDR-H1. The C8 clone was the dominant clone among the selected binders, suggesting preferential enrichment during the selection process. Competitive ELISA assays demonstrated reduced phage binding following pre-incubation with soluble Stx1, indicating epitope saturation and suggesting specific antigen recognition (Figure 1A). Cross-reactivity assays further confirmed that both FabB6:Stx1 and FabC8:Stx1 were specific for Stx1 and did not bind to Stx2 (Figure 1B), highlighting their selective recognition of Stx1 epitopes.

Therefore, the genes encoding FabB6:Stx1 and FabC8:Stx1 were amplified, cloned into an expression vector, and successfully expressed in *E. coli* BL21(DE3) pLysS. Successful cloning was confirmed by restriction analysis with NsiI and SalI, which revealed the expected bands corresponding to the vector backbone and the Fab insert (Figure S1). Both Fabs were purified to homogeneity, and SDS-PAGE under reducing and non-reducing conditions verified their expected molecular weights and proper structural assembly (Figure S2).

### 2.2. Binding Affinity and Functional Characterization

**Figure 2:**
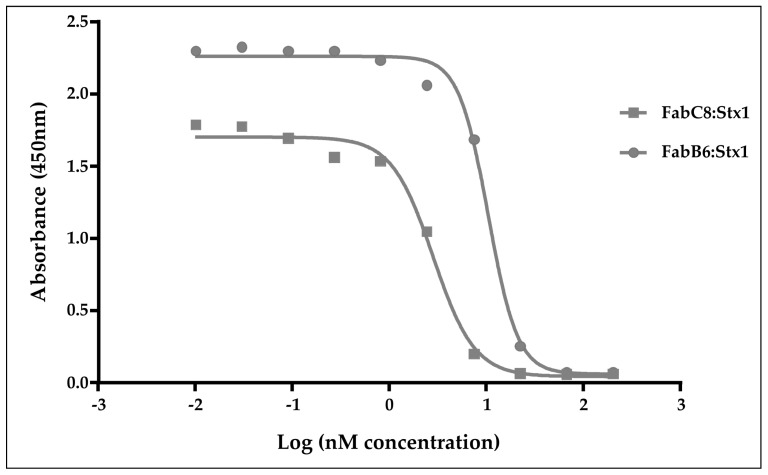
Determination of the half-maximal effective concentration (EC_50_) of FabB6:Stx1 and FabC8:Stx1 by ELISA. The binding efficiency of the purified Fabs to immobilized Stx1 was evaluated using serial dilutions of each antibody. The absorbance at 450 nm is plotted against the log of the Fab concentration in nM. FabC8:Stx1 exhibited a higher apparent binding affinity (EC50 = 50 ng/mL) than FabB6:Stx1 (EC50 = 160 ng/mL). Data points represent the mean ± SD of triplicate measurements, and curves were generated by nonlinear regression analysis.

The binding properties of FabB6:Stx1 and FabC8:Stx1 were further characterized using ELISA-based EC_50_ assays and surface plasmon resonance (SPR). FabB6:Stx1 exhibited an EC_50_ of 160 ng/mL, whereas FabC8:Stx1 showed higher apparent binding efficiency, with an EC_50_ of 50 ng/mL (Figure 2). SPR analyses revealed nanomolar affinities for both antibodies, with calculated dissociation constants (KD) of approximately 40 nM for FabB6:Stx1 and 10 nM for FabC8:Stx1, indicating a higher affinity of FabC8:Stx1 for Stx1. SPR data are presented in Table 1.

### 2.3. Neutralization of STEC Strains-Derived Stx1 In Vero and Human Proximal Tubular Epithelial (HK-2) Cells

**Figure 3:**
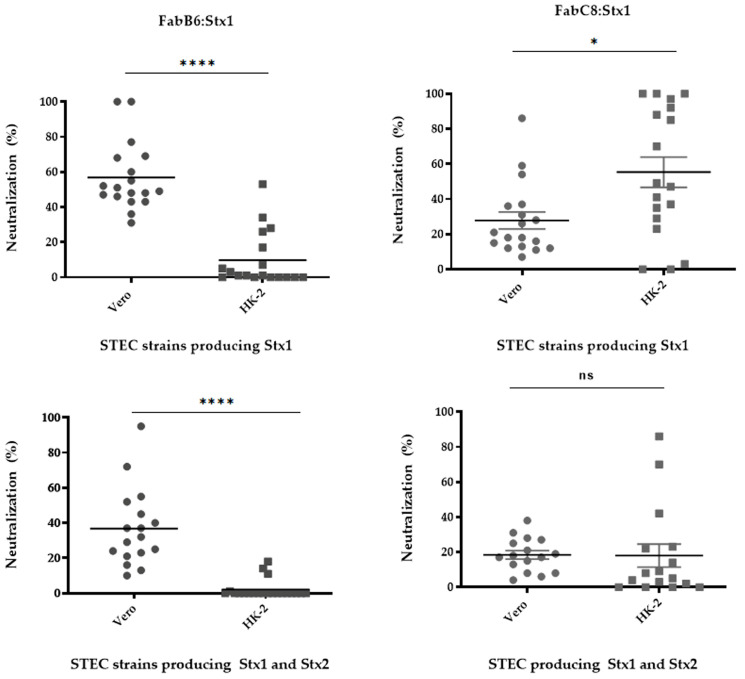
Percentage of neutralization of FabB6:Stx1 and FabC8:Stx1. Fabs were preincubated with STEC culture supernatants producing Stx1 or Stx1 and Stx2 toxins (diluted 1/50) and were exposed to Vero (closed circle) and HK-2 (closed square) cells. The ability of neutralization was assessed 72 h post-treatment using the crystal violet assay. Data represent the mean ± SD of three independent experiments. Statistical significance was determined using the one-way Wilcoxon matched-pairs signed-rank test. Differences were considered statistically significant at *p* < 0.05. **** *p* < 0.0001, VERO vs. HK-2 (neutralization % of STEC strains producing Stx1 and Stx1/Stx2 by FabB6:Stx1); * *p* < 0.05, HK-2 vs. VERO (neutralization % of STEC strains producing Stx1 by FabC8:Stx1). ns: not significant.

The neutralizing activity of FabB6:Stx1 and FabC8:Stx1 against Stx1-containing STEC culture supernatants was evaluated in Vero and HK-2 cells. Overall, FabB6:Stx1 showed a stronger neutralization profile in Vero cells, with inhibition values frequently ranging between ~40–100%, whereas its neutralizing capacity in HK-2 cells was markedly reduced, often approaching minimal levels. In contrast, FabC8:Stx1 displayed a distinct pattern, showing moderate or low neutralization in Vero cells but markedly higher inhibitory activity in HK-2 cells, in several cases reaching nearly complete neutralization. This difference was particularly evident for strains such as H19, 3529, 82, and EPM11, in which FabC8:Stx1 achieved high levels of protection in HK-2 cells despite relatively modest inhibition in Vero cells (Table 2; Figure 3).

These results suggest that FabB6:Stx1 and FabC8:Stx1 recognize distinct epitopes or interfere differently with toxin–cell interactions, leading to cell-type-dependent neutralization profiles. Notably, strains harboring both *stx1* and *stx2* genes generally exhibited reduced neutralization by both antibodies, indicating that residual cytotoxicity in these cases is likely mediated by Stx2, which is not targeted by the anti-Stx1 Fab fragments. The complementary neutralization patterns observed for FabB6:Stx1 and FabC8:Stx1 further suggest that combining antibodies targeting different epitopes may enhance overall protection against Stx-mediated cytotoxicity.

### 2.4. Cytotoxicity of HUS-Derived STEC Supernatants in Human Glomerular Endothelial Cells (HGECs)

**Figure 4:**
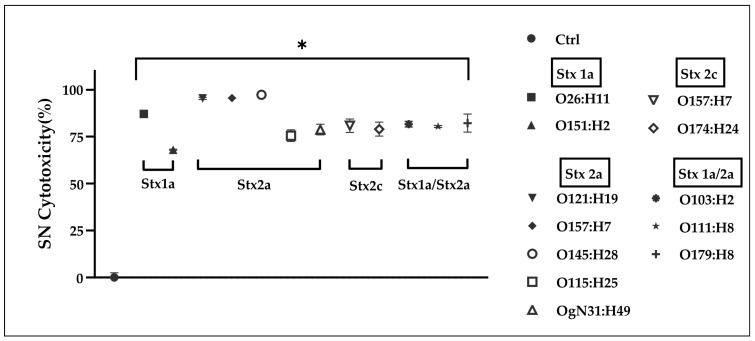
Cytotoxicity of STEC clinical isolates supernatants (STEC-SN) in HGEC. Cells were exposed to culture supernatants from STEC strains producing Stx1a, Stx2a, Stx2c, or both Stx1a and Stx2a. Cell viability was assessed after 72 h. Data represent the mean ± SEM of three independent experiments. Statistical significance was determined by one-way ANOVA followed by Tukey’s post hoc test (* *p* < 0.05, STEC-SN vs. control [Ctrl]). Table 3. STEC strains isolated from HUS patients: characteristics and cytotoxicity on HGECs. Stx supernatant concentration was estimated with the Stx1a, Stx2a, or Stx2a + Stx1a standard curves.

Before the neutralization assays, the intrinsic cytotoxicity of STEC strains isolated from HUS patients was evaluated in primary HGEC cultures. All tested strains induced pronounced cytotoxic effects, irrespective of Stx subtype, confirming the high susceptibility of HGEC to STEC-derived toxins and supporting their relevance as a physiologically meaningful model of endothelial injury associated with HUS. In addition, Stx subtype concentration in each STEC supernatant was estimated by interpolating the corresponding HGEC % viability on standard curves for purified Stx (Figure 4; Table 3), allowing a comparative assessment of toxin levels across different STEC strains.

### 2.5. Neutralization of HUS-Derived STEC Supernatants in HGEC by Recombinant Fabs

**Figure 5:**
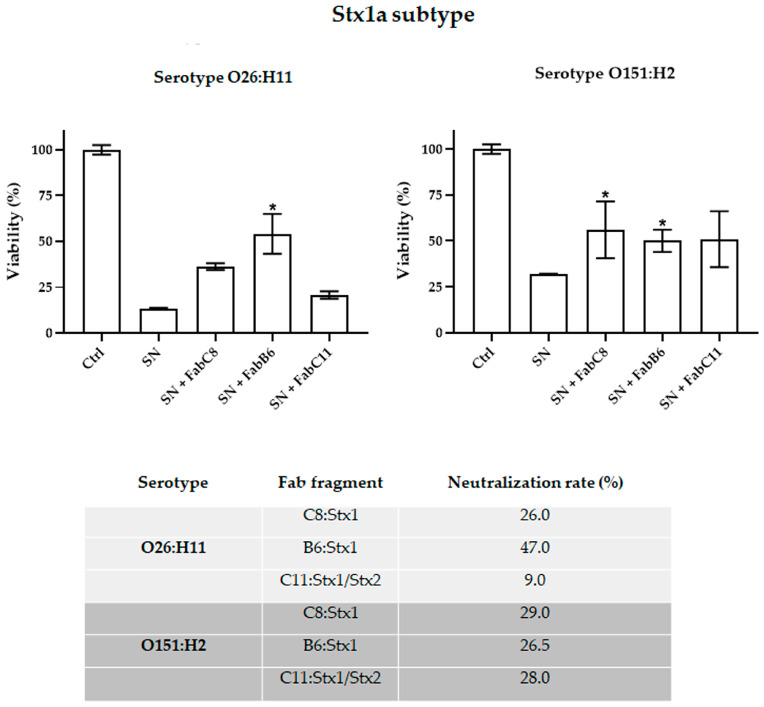
Neutralization of Stx1a-producing STEC isolates in HGEC. (**Upper panels**) Cell viability was assessed after 72 h of exposure to culture supernatants from clinical isolates expressing Stx1a (O26:H11 and O151:H2) in the presence of FabB6:Stx1, FabC8:Stx1, or FabC11:Stx1/Stx2. Bars represent the mean percentage of viability ± SEM of three independent experiments performed in duplicate. Statistical significance was evaluated by one-way ANOVA with Tukey’s post hoc test. (* *p* < 0.05, Fab-treated vs. SN). (**Lower panel**) The embedded table details the corresponding Neutralization rate (%), calculated based on the viability data, where 100% represents the complete reversal of STEC-SN cytotoxic effects to control levels.

The neutralizing capacity of FabB6:Stx1 and FabC8:Stx1 was subsequently evaluated in HGECs using the culture supernatant of STEC isolates expressing Stx1 either alone or in combination with Stx2. For Stx1a-producing strains, both FabB6:Stx1 and FabC8:Stx1 conferred partial yet consistent protection, with neutralization efficiencies varying among isolates (Figure 5).

**Figure 6:**
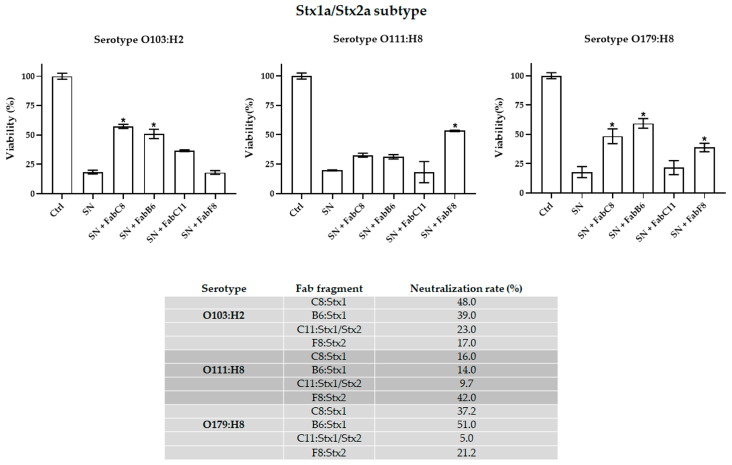
Neutralization of Stx1a/Stx2a-producing STEC isolates in HGEC. (**Upper panels**) Cell viability was assessed after 72 h of exposure to culture supernatants from clinical isolates co-expressing Stx1a and Stx2a (O103:H2, O111:H8, and O179:H8). The assay compares the neutralizing capacity of individual anti-Stx1 Fabs (FabB6:Stx1 and FabC8:Stx1) with the pre-characterized anti-Stx2 FabF8:Stx2, and the cross-reactive FabC11:Stx1/Stx2. Bars represent the mean percentage of viability ± SEM of three independent experiments performed in duplicate. Statistical significance was determined by one-way ANOVA followed by Tukey’s post hoc test (* *p* < 0.05, Fab-treated vs. SN). (**Lower panel**) The embedded table summarizes the calculated Neutralization rate (%) for each treatment, defining 100% neutralization as the full restoration of cell viability relative to untreated controls.

For strains co-expressing Stx1a and Stx2a, FabB6:Stx1 and FabC8:Stx1 improved neutralization in specific isolates (O103:H2 and O179:H8), whereas FabF8:Stx2 was more effective against O111:H8 (Figure 6). This complementary pattern indicates that the relative contribution of each toxin to cytotoxicity varies among isolates.

**Figure 7:**
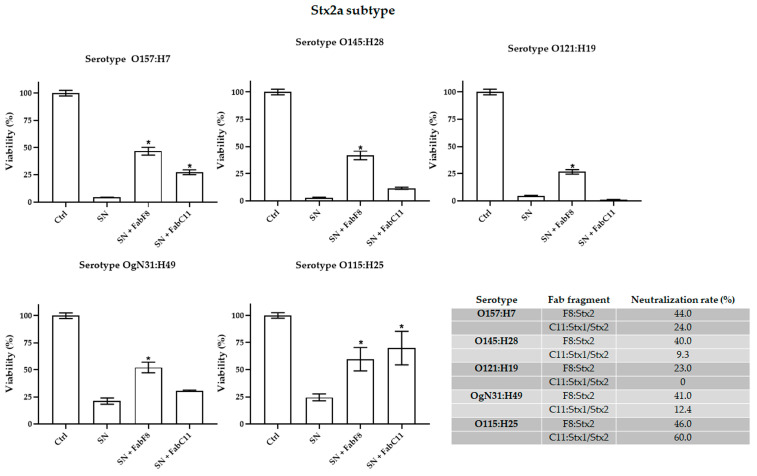
Neutralization of Stx2a-producing STEC clinical isolates by FabF8:Stx2 and FabC11:Stx1/Stx2, in HGEC. Cell viability was determined 72 h post-exposure to culture supernatants from isolates expressing the Stx2a subtype (O121:H19, O157:H7, O145:H28, OgN31:H49, and O115:H25). Data represent the mean percentage of cell viability ± SEM from three independent experiments. Statistical significance was assessed using one-way ANOVA followed by Tukey’s post hoc test (* *p* < 0.05). The embedded table provides the Neutralization rate (%), derived from the viability data, where 100% neutralization indicates a complete reversal of the STEC-SN cytotoxic effect to control levels.

**Figure 8:**
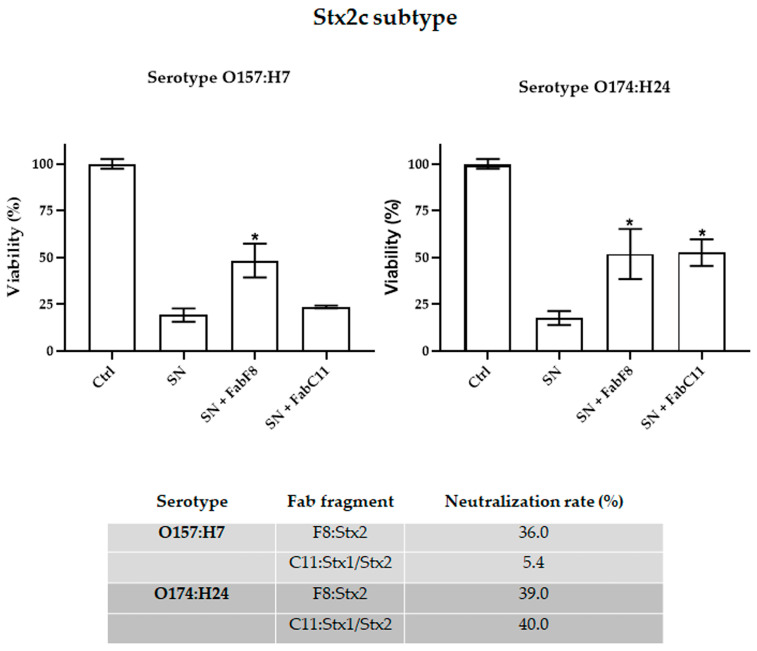
Neutralization of Stx2c-producing STEC clinical isolates in HGEC. (**Upper panels**) Cells were treated with culture supernatants from isolates expressing the Stx2c subtype (O157:H7 and O174:H24). The assay evaluates the neutralizing capacity of FabF8 and FabC11 following a 72 h incubation period. Bars indicate the mean ± SEM of triplicate measurements from three independent assays. Statistical significance was assessed using one-way ANOVA followed by Tukey’s post hoc test (* *p* < 0.05). (**Lower panel**) The embedded table displays the calculated Neutralization rate (%) for the respective treatments, defined by the percentage of cell viability restored relative to the untreated controls.

For assays evaluating exclusively Stx2 producing strains, FabC11:Stx1/Stx2 and FabF8:Stx2 were incorporated not merely as reference controls, but to conduct the first evaluation of these Fab fragments against HUS-derived clinical strains. While FabF8:Stx2 exhibited superior neutralization of Stx2a- and Stx2c-producing strains, FabC11:Stx1/Stx2 still afforded substantial protection in specific cases (Figure 7 and Figure 8), thereby corroborating the primary role of Stx2 in the cytotoxicity of these isolates. Collectively, these results establish that a strategic combination of Stx1- and Stx2-targeted recombinant Fabs substantially broadens the neutralization spectrum against highly diverse clinical STEC isolates.

**Figure 9:**
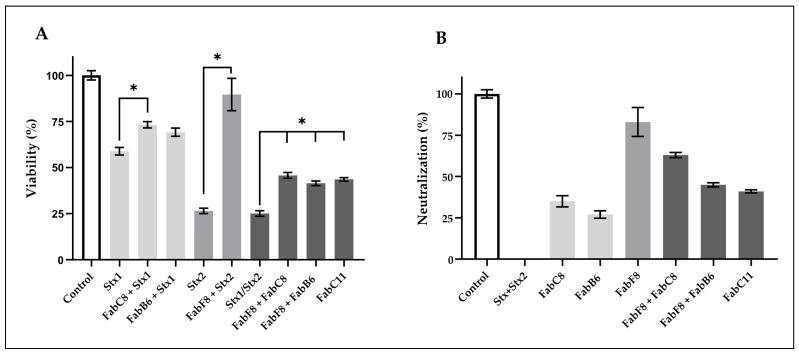
Neutralization assays employing purified Stx1 and/or Stx2. The assay evaluates the neutralizing capacity of Fabs individually or in combination. (**A**). Percentage of HGECs’ viability in the presence of Stx or pre-incubating with Fabs. (**B**). Percentage of Stx neutralization by Fabs. Bars indicate the mean ± SEM of triplicate measurements from three independent assays. Statistical significance was assessed using one-way ANOVA followed by Tukey’s post hoc test (* *p* < 0.05, Stx vs. Ctrl).

To investigate combinatorial neutralization efficacy, Fab mixtures (FabC8 + FabF8 and FabB6 + FabF8), together with the cross-reactive FabC11, were evaluated against the simultaneous presence of Stx1 and Stx2. Cell viability and neutralization efficiency were subsequently determined (Figure 9A,B). At 0.5 ng/mL, Stx2 induced substantially greater cytotoxicity in HGEC than Stx1, while the combination of both toxins induced cytotoxicity comparable to that caused by Stx2 alone, highlighting the predominant contribution of Stx2 to overall toxicity. Consistently, pre-incubation with either FabC8 + FabF8 or FabB6 + FabF8 resulted in markedly higher protection than treatment with the Stx1-specific Fabs alone, largely due to the potent neutralizing activity of FabF8 against Stx2. Nevertheless, these findings support the therapeutic advantage of simultaneously targeting both toxins to achieve broader protective coverage. Importantly, the protection mediated by the Fab combinations was comparable to that achieved with the cross-reactive FabC11.

## 3. Discussion

This study expands the therapeutic repertoire against Shiga toxin-producing *Escherichia coli* (STEC) by introducing two novel, fully human Fabs, FabB6:Stx1 and FabC8:Stx1, specifically targeting Stx1. While much of the recent literature has focused on Stx2 due to its strong association with severe clinical outcomes [20,21,22], the persistent prevalence of Stx1-producing strains, either alone or in combination with Stx2, highlights a critical gap in current anti-toxin strategies. Our results demonstrate that FabB6:Stx1 and FabC8:Stx1 possess nanomolar affinity for Stx1 and provide significant protection across various cellular models relevant to STEC pathogenesis.

The distinct neutralization profiles observed for FabB6:Stx1 and FabC8:Stx1 in Vero and HK-2 cells likely reflect differences in toxin–receptor interactions and epitope accessibility on the Stx1 holotoxin [5,23,24,25]. Vero cells are widely used for Shiga toxin cytotoxicity assays due to their high expression of the Gb3 receptor, which facilitates toxin binding and internalization. However, HK-2 cells represent a more physiologically relevant model of human renal proximal tubular epithelium, a primary target tissue during hemolytic uremic syndrome (HUS) [25,26,27]. In this context, Lenz et al. demonstrated the differential susceptibility of HK-2 and Vero cells to Stx1-mediated cytotoxicity [28]. The markedly greater neutralization achieved by FabC8:Stx1 in HK-2 cells suggests that this antibody may more effectively interfere with toxin binding or internalization in renal epithelial cells. These differences may be related to the specific epitope recognized by FabC8:Stx1, which may more effectively interfere with toxin–receptor interactions or sterically hinder Gb3 binding mediated by the toxin B subunits. Notably, although FabC8:Stx1 exhibited higher binding affinity, affinity alone does not necessarily correlate with neutralization potency, indicating that factors such as epitope specificity and cellular context likely contribute to antibody performance.

Neutralizing antibodies against Stx1 predominantly target the B subunit, thereby preventing binding to the Gb3 receptor and subsequent toxin internalization. Classical monoclonal antibodies, such as the chimeric cαStx1 derived from 13C4 and other anti-Stx1 mAbs, have demonstrated that interference with receptor engagement is sufficient to abrogate cytotoxicity in vitro and protect against toxin challenge in vivo [15,16,17,18]. Similarly, antibody 5-5B neutralizes Stx1 by blocking receptor binding, reinforcing the central role of the B subunit in mediating toxicity [19]. Consistent with this mechanism, our recombinant Fabs, FabB6:Stx1 and FabC8:Stx1, were selected against Stx1 and effectively neutralized toxin-induced cytotoxicity in epithelial and endothelial cell models. Differences in neutralization profiles across cell types suggest that epitope specificity and affinity may influence the efficiency of receptor blockade and/or intracellular trafficking. Importantly, FabC11:Stx1/Stx2 extends this mechanism by recognizing conserved epitopes shared between Stx1 and Stx2, enabling cross-neutralization and highlighting functionally constrained regions of the toxin.

Furthermore, the complementary neutralization profiles observed for FabB6:Stx1 and FabC8:Stx1 support the notion that antibodies targeting distinct epitopes may act synergistically or complementarily to enhance overall protection. This observation highlights the potential advantage of antibody combinations or cocktails for improving the neutralization breadth against Shiga toxins and reducing toxin-mediated cytotoxicity in clinically relevant cell types.

Importantly, when tested in primary human glomerular endothelial cells (HGEC) using STEC isolates from HUS patients, FabB6:Stx1 and FabC8:Stx1 provided partial but reproducible protection against Stx1-mediated cytotoxicity. The marked intrinsic susceptibility of HGEC to STEC-derived toxins underscores the clinical relevance of this cellular model while emphasizing the challenge of achieving complete toxin neutralization in a highly sensitive target tissue. According to this, we have not yet been able to achieve Shiga toxin complete neutralization by Fab on HGEC [13,14]. Notably, the combined evaluation of Stx1- and Stx2-specific fragments revealed a complementary pattern: FabF8:Stx2 and FabC11:Stx1/Stx2 provided superior protection against Stx2-producing strains, while FabB6:Stx1 and FabC8:Stx1 contributed essential protection against Stx1-containing isolates.

The marked variability in neutralization efficiency observed among STEC strains producing identical Stx subtypes further emphasizes that toxin-mediated cytotoxicity is modulated by multiple strain-specific factors, including toxin expression levels, phage induction dynamics, and the presence of ancillary virulence determinants [29,30]. Collectively, these observations reinforce the emerging paradigm that effective therapeutic strategies against STEC-associated disease are unlikely to rely on a single antibody specificity, but instead may require combinatorial approaches capable of simultaneously targeting distinct toxins and functionally relevant epitopes to achieve broader and more robust protection. While these findings provide important insights, some limitations should be considered, including the lack of in vivo validation and the absence of epitope characterization, which limits a deeper mechanistic understanding of Fab-mediated neutralization and its translation to in vivo settings, albeit the consistency of the observed neutralization patterns across different experimental models supports the robustness of the present findings.

Altogether, these findings provide evidence that Shiga toxin neutralization exhibits toxin- and cell-type dependency, furthering the argument that human-derived renal systems represent a critical platform for translational validation. The integration of Stx1-specific recombinant Fabs alongside established anti-Stx2 antibodies significantly broadens the neutralization spectrum against diverse STEC isolates. In summary, these findings support a multi-target neutralization strategy as a rational and versatile platform for the development of next-generation immunotherapeutic or adjunctive approaches aimed at limiting toxin-mediated renal and endothelial injury in STEC-associated diseases.

## 4. Materials and Methods

### 4.1. Bacterial Strains, Plasmids, and Antigen

Phage-resistant *Escherichia coli* Omnimax (Invitrogen, Waltham, MA, USA) was used for phage display selections. For cloning and expression of Fabs, *E. coli* DH5α (Thermo Fisher Scientific, Waltham, MA, USA) and BL21 (DE3) pLysS (Novagen, Madison, WI, USA) strains were used, respectively. The phagemid vector HP153 and the expression vector pFab-MBP were employed as previously described [12]. A panel of STEC strains analyzed in this study comprises 18 strains harboring the *stx1* gene and 16 harboring the *stx1* and *stx2* genes from human and animal feces [31] (Table 1). In addition, strains isolated from HUS patients and previously defined as STEC by gene presence and Stx1 or Stx2 production were provided by the Servicio Fisiopatogenia, Departamento Bacteriología, Instituto Nacional de Enfermedades Infecciosas ANLIS “Dr. Carlos G. Malbrán”, Buenos Aires, Argentina (Table 2), including the prototype EDL933 (O157:H7) [32], since the focus was to analyze Stx produced by strains isolated from HUS patients. Purified Stx2a and Stx1a were obtained from Phoenix Laboratory, Tufts Medical Center, Boston, MA, USA.

### 4.2. Selection, Expression, and Purification of Recombinant Fabs

FabB6:Stx1 and FabC8:Stx1 were selected from a human synthetic Fab phage-display library [33] using immobilized Stx1 as antigen. Four rounds of biopanning were performed following established protocols [34]. Selected clones were Sanger sequenced, and Fab-encoding genes were cloned into expression vectors using the NsiI and SalI restriction enzyme sites. The same restriction enzymes were used to confirm successful cloning by restriction analysis. Recombinant Fabs were expressed in *E. coli* BL21 (DE3) pLysS and purified by affinity chromatography, as previously described. FabC11:Stx1/Stx2 and FabF8:Stx2 were produced in parallel for comparative neutralization assays [12,13,14].

### 4.3. Binding and Affinity Analyses

The binding specificity of Fabs was assessed by ELISA using purified Stx1 and Stx2. Half-maximal effective concentrations (EC_50_) were determined by serial dilution. Binding kinetics and affinities were measured by surface plasmon resonance using a Biacore T200 system, applying a 1:1 Langmuir binding model. The employed methodology was described in detail in Luz et al. (2021) [14].

### 4.4. Cell Lines and Culture Conditions

Vero cells were cultured in Dulbecco’s modified Eagle medium (DMEM) supplemented with 10% fetal bovine serum (FBS). Human proximal tubular epithelial cells (HK-2) were cultured in DMEM/F12 supplemented with 5% FBS, insulin–transferrin–selenium, hydrocortisone, and antibiotics. Primary human glomerular endothelial cells (HGECs) were isolated from pediatric kidney tissue under approved ethical protocols and cultured in Medium 199 supplemented with 20% fetal calf serum and 25 g/mL endothelial growth supplement. HGEC between passages 2 and 7 was used in all experiments [27].

### 4.5. HGEC Viability Assay

STEC supernatant (STEC-SN) cytotoxicity was evaluated using the neutral red viability assay, as previously described [27]. HGEC were seeded in 96-well plates and grown to confluence in complete medium. Cells were then exposed to filtered STEC culture supernatants diluted 1:50 from strains harboring *stx1*, *stx2*, or both *stx1*/*stx2* genes, or to different concentrations of purified Stx1a, Stx2a, or combined Stx1a + Stx2a. The growth-arrested culture medium was used as a control. Subsequently, freshly prepared neutral red solution (Sigma-Aldrich, St. Louis, MO, USA) was added to a final concentration of 10 μg/mL, and cells were incubated for an additional 1 h at 37 °C in 5% CO_2_. The HGEC were then washed and fixed with 200 mL 1% CaCl_2_ / 1% formaldehyde, and then lysated with 200 mL 1% acetic acid in 50% ethanol, where neutral red was solubilized. Absorbance in each well was measured in an automated plate spectrophotometer at 540 nm. To establish the cell viability percentage, 100% was assigned to the control cells, which were incubated under identical conditions but without treatment. Results were expressed as percentage cytotoxicity, with control cells defined as 0% cytotoxicity. To estimate the Stx subtype concentration in each STEC-SN, the corresponding HGEC % viability was interpolated on standard curves for purified Stx1a, Stx2a, and Stx1a + Stx2a.

### 4.6. Neutralization Assays

Neutralization assays were performed using STEC-derived supernatants and purified Shiga toxins as a control. Fabs were pre-incubated with purified toxins or STEC supernatants prior to their addition to the cell monolayers. Vero and HK-2 cell viability was assessed by MTT assay, whereas HGEC viability was evaluated using the neutral red uptake assay [27]. All assays were performed in duplicate and repeated in at least three independent experiments. Complete neutralization (100%) was defined as the total reversal of STEC-SN-induced cytotoxic effects.

### 4.7. Experimental Design for Fabs Neutralization Assay Using STEC Isolates Supernatant

STEC bacterial supernatants were obtained as described by Shiga et al. 2020 [35]. HGECs (1 × 10^5^ cells/mL) were seeded to confluence in 96-well plates at 37 °C in a humidified atmosphere in 5% CO_2_ for 24 h. The neutralizing activity of FabB6:Stx1, FabC8:Stx1, FabF8:Stx2, and FabC11:Stx1/Stx2 was evaluated by pre-incubating STEC bacterial supernatants (diluted 1:50) with an equal volume of Fab at 10 µg/mL. Pre-incubations were carried out at growth-arrested conditions by using M199 medium supplemented with 10% FBS at 37 °C for 1 h. The mixtures were then added to HGECs and incubated for an additional 72 h at 37 °C in 5% CO_2_. HGEC in arrest medium (controls: Ctrl) and STEC supernatant non-pre-incubated with Fab (SN) were used as controls. All assays were performed in duplicate and independently repeated three times.

### 4.8. Statistical Analysis

Data are presented as mean ± standard deviation (SD) and mean ± standard error of the mean (SEM). Statistical analyses were performed using Student’s *t*-test or one-way analysis of variance (ANOVA) followed by Tukey’s multiple-comparison test. A *p*-value < 0.05 was considered statistically significant.

## Figures and Tables

**Table 1:** FabB6:Stx1 and FabC8:Stx1 calculated parameters by SPR.

Sample	Ka (1/Ms)	Kd (1/s)	Kd (M)	Chi^2^ (RU^2^)	Ligand	Model
FabB6:Stx1	3.12 × 10^4^	1.25 × 10^−3^	3.99 × 10^−8^	1.02	Stx1 5 µg/mL	1:1 Binding
FabC8:Stx1	9.54 × 10^4^	1.75 × 10^−3^	1.83 × 10^−8^	0.749	Stx1 5 µg/mL	1:1 Binding

**Table 2:** Culture supernatant sources of STEC strains expressing Stx1 and Stx1/Stx2 characteristics and percentage of neutralization (%) by FabB6:Stx1 and FabC8:Stx1 to Stx1 in Vero and HK-2 cells.

Strains	Serotype	Source	Stx	Subtype	Neutralization % by FabB6:Stx1		Neutralization % by FabC8:Stx1	
					Vero	HK-2	Vero	HK-2
**BA 597**	OR:NM	Human	Stx1	1a	69	53	28	0
**BA 4123**	O26:H11	Human	Stx1	1a	77	26	86	49
**D360-4-1**	O26:H11	Animal	Stx1	1a	100	1.1	21	0
**1557-77**	O26:H11	Human	Stx1	1a	48	0	31	37
**H30**	O26:H11	Human	Stx1	1a	100	0	59	41
**H19**	O26:H11	Human	Stx1	1a	49	34	54	100
**199**	O26:H11	Human	Stx1	1a	43	1.5	7	29
**3529**	O26:H11	Human	Stx1	1a	60	3.5	37	98
**82**	O157:H7	Unknown	Stx1	1a	46	0	12	100
**3299-85**	O157:H7	Human	Stx1	1a	36	1	36	83
**3077-88**	O157:H7	Human	Stx1	1a	68	7	15	47
**EPM 5**	O55:H19	Human	Stx1	1a	31	0	26	70
**EPM 11**	O118:H16	Human	Stx1	1a	51	0	18	100
**EPM 16**	O26:H11	Human	Stx1	1a	43	17	11	88
**EPM 20**	O111:H8	Human	Stx1	1a	47	0	13	85
**EPM 26**	O111:NM	Human	Stx1	1a	48	0	18	3
**EPM 27**	O111:NM	Human	Stx1	1a	52	28	12	35
**EPM O17**	O112:H2	Animal	Stx1	1 c	55	5	16	92
**BA 3003**	O48:H7	Human	Stx1/2	1a, 2a	45	14	31	14
**3104-88**	O157:H7	Human	Stx1/2	1a, 2a	21	0	15	0
**18 (ICB)**	Unknown	Unknown	Stx1/2	UND	25	0	8	2
**EPM 4**	O93:H19	Human	Stx1/2	1a, 2b	55	18	17	70
**EPM 9**	O103:H2	Human	Stx1/2	1a, 2c	52	0	21	42
**EPM 23**	O111:H8	Human	Stx1/2	1a	95	0	13	86
**EPM 44**	O98:H4	Animal	Stx1/2	1a, 2NT	32	0	27	8
**EPM 45**	O181:H4	Animal	Stx1/2	1a, 2a	29	0	38	0
**EPM 53**	O98:H17	Animal	Stx1/2	1a, 2a, 2c	23	0	6	0
**EPM 55**	O98:H17	Animal	Stx1/2	1a, 2a, 2c	72	1	18	23
**EPM 66**	O105:H18	Animal	Stx1/2	1a, 2a, 2b	13	0	4	9
**EPM 81**	ONT:H38	Animal	Stx1/2	1NT, 2a	37	0	25	4
**EPM O1**	ONT:H8	Animal	Stx1/2	UND	37	0	17	22
**EPM O36**	O75:H8	Animal	Stx1/2	1c, 2b	40	11	19	0
**EPM O55**	O146:H21	Animal	Stx1/2	1a, 2a, 2b	24	0	8	5
**EDL 933**	0157:H7	Food	Stx1/2	1a, 2a	16	0	28	4

**Table 3:** STEC strains isolated from HUS patients: characteristics and cytotoxicity on HGECs.

Serotype	Stx Subtype	Cytotoxicity (%)	Stx Supernatant Concentration (μg/mL) *
O26:H11	Stx1a	87	3.7
O151:H2	Stx1a	68	0.6
O157:H7	Stx2a	95.6	>5.0
O145:H28	Stx2a	97.3	>5.0
O121:H19	Stx2a	95.4	4.9
OgN31:H49	Stx2a	78.8	0.05
O115:H25	Stx2a	75.5	0.04
O103:H2	Stx1a/Stx2a	81.6	0.14
O111:H8	Stx1a/Stx2a	80.4	0.12
O179:H8	Stx1a/Stx2a	82.2	0.15
O157:H7	Stx2c	80.8	Not established
O174:H24	Stx2c	79.0	Not established

* The Stx1 or Stx2 standard curve determined the Stx supernatant concentration.

## Data Availability

The original contributions presented in this study are included in the article/Supplementary Materials. Further inquiries can be directed to the corresponding authors.

## Supplementary Materials

The following supporting information can be downloaded at: https://www.mdpi.com/article/10.3390/toxins18060257/s1, Figure S1: Agarose gel electrophoresis (1.5%) stained with SYBR Green (1:1000) showing restriction analysis of Fab clones. Lane 1, 1 kb DNA ladder (Invitrogen); lane 2, anti-Stx1 Fab clone B6; lane 3, anti-Stx1 Fab clone C8; Figure S2: SDS–PAGE analysis (12%) of purified Fabs stained by silver nitrate. Eluted samples are shown under reducing in presence of Dithiothreitol-DTT: (+DTT) and non-reducing (−DTT) conditions. Lane 1, Blueyed molecular weight marker (10–180 kDa GE); lane 2, anti-Stx1 Fab clone B6 (−DTT); lane 3, anti-Stx1 Fab clone C8 (−DTT); lane 4, anti-Stx1 Fab clone B6 (+DTT); lane 5, anti-Stx1 Fab clone C8 (+DTT).
