# Supplementary material for: Recombinant Human Fab Antibodies Differentially Neutralize Shiga Toxin in Renal Epithelial and Endothelial Cells
Source: Toxins (Basel). 2026 Jun 5;18(6):257. doi: 10.3390/toxins18060257 (PMC13307652; doi:10.3390/toxins18060257)
Supplement: Supplementary file 1 [file toxins-18-00257-s001.zip › toxins-4304223-supplementary.pdf]

Supplementary Materials: Recombinant Human Fab Antibodies Differentially Neutralize Shiga Toxin in Renal Epithelial and Endothelial Cells

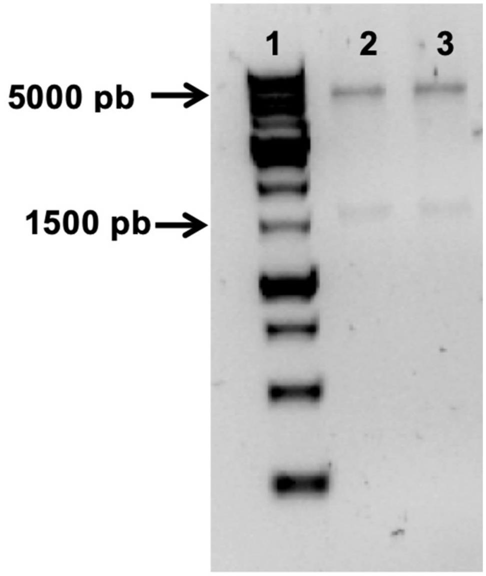

Figure S1. Agarose gel electrophoresis (1.5%) stained with SYBR Green (1:1000) showing restriction analysis of Fab clones. Lane 1, 1 kb DNA ladder (Invitrogen); lane 2, anti-Stx1 Fab clone B6; lane 3, anti-Stx1 Fab clone C8.

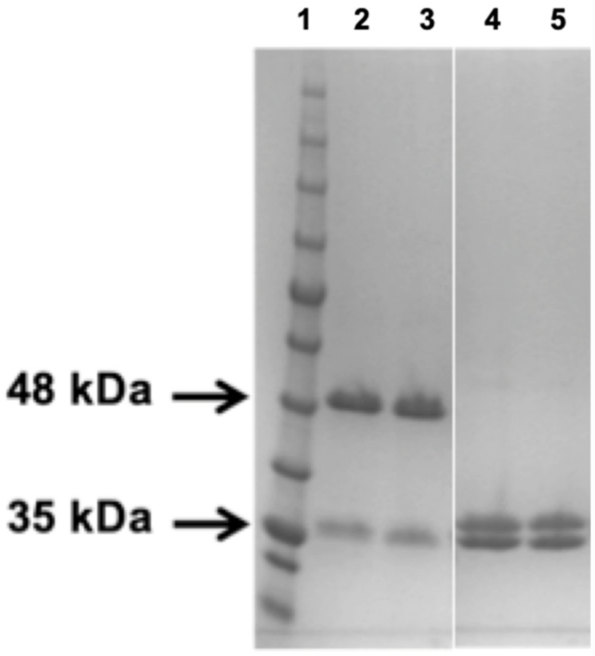

Figure S2. SDS–PAGE analysis (12%) of purified Fabs stained by silver nitrate. Eluted samples are shown under reducing in presence of Dithiothreitol-DTT: (+DTT) and non-reducing (−DTT) conditions. Lane 1, Blueyed molecular weight marker (10-180 kDa GE); lane 2, anti-Stx1 Fab clone B6 (-DTT); lane 3, anti-Stx1 Fab clone C8 (-DTT); lane 4, anti-Stx1 Fab clone B6 (+DTT); lane 5, anti-Stx1 Fab clone C8 (+DTT).
